# Imaging Assessment of Interval Metastasis from Melanoma

**DOI:** 10.3390/jpm12071033

**Published:** 2022-06-24

**Authors:** Igino Simonetti, Piero Trovato, Vincenza Granata, Carmine Picone, Roberta Fusco, Sergio Venanzio Setola, Mauro Mattace Raso, Corrado Caracò, Paolo A. Ascierto, Fabio Sandomenico, Antonella Petrillo

**Affiliations:** 1 Radiology Division, Istituto Nazionale Tumori-IRCCS-Fondazione G. Pascale, 80131 Napoli, Italy; igino.simonetti@istitutotumori.na.it (I.S.); c.picone@istitutotumori.na.it (C.P.); s.setola@istitutotumori.na.it (S.V.S.); m.mattaceraso@istitutotumori.na.it (M.M.R.); a.petrillo@istitutotumori.na.it (A.P.); 2 Radiology Division, “ASL Napoli II Nord”, 33939 Naples, Italy; piero_trovato@hotmail.it; 3 Medical Oncology Division, Igea SpA, 80013 Napoli, Italy; r.fusco@igeamedical.com; 4 Italian Society of Medical and Interventional Radiology (SIRM), SIRM Foundation, 20122 Milan, Italy; 5 Melanoma, Cancer Immunotherapy and Development Therapeutics Unit, Istituto Nazionale Tumori-IRCCS-Fondazione G. Pascale, 80131 Naples, Italy; corrado.caraco@istitutotumori.na.it (C.C.); p.ascierto@istitutotumori.na.it (P.A.A.); 6 Radiology Division, Ospedale Fatebenefratelli, 80123 Naples, Italy; f.sandomenico@istitutotumori.na.it

**Keywords:** melanoma, ultrasound, computed tomography, magnetic resonance imaging, positron emission tomography

## Abstract

Interval metastasis is a particular metastatic category of metastatic localizations in the lymph nodes in patients with melanoma. Interval nodes are generally located at nonregional lymphatic stations placed along the pathway of the spread of melanoma, such as the epitrochlear lymph node station, the popliteal fossa, and the retroareolar station. Imaging techniques for evaluation of patients with interval metastasis from melanoma diseases include ultrasound (US), computed tomography (CT), magnetic resonance imaging (MRI), lymphoscintigraphy (LS), and positron emission tomography (PET). A literature review was conducted through a methodical search on the Pubmed and Embase databases. The evaluation of lymph node metastases represents a critical phase in the staging and follow-up of melanoma patients. Therefore, a thorough knowledge of the imaging methods available and the interactions between the clinician and the radiologist are essential for making the correct choice for individual patients, for a better management, and to improve treatment and survival.

## 1. Introduction

Melanoma accounts for approximately 1% of all skin cancers diagnosed, with a global number of new cases in 2020 of 324,635 and a death toll of 57,043. The highest incidence is in Europe (150,627—46.4%), followed by Northern America (105,172—32.4%) and Asia (23,753—7.3%), with death rates in Europe of 26,360 (46.2%), in Asia of 11,986 (21%) and in Northern America of 8412 (14.7%) [1]. 

Although melanoma represents only a minority of all skin cancers, this tumor type causes the majority of skin-cancer-related deaths worldwide [1]. In this scenario, an early diagnosis and accurate treatment should improve outcomes and survival [1]. However, today, there are still a significant number of patients who present with or later develop loco-regional or distant recurrence [1]. These patients require ongoing management, and for them, an accurate risk assessment remains an open but critical and key question [1].

Current melanoma treatments include multidisciplinary approaches that involve surgery, chemotherapy, and radiotherapy. Nevertheless, with the exception of those with early-stage disease, patients typically have poor prognoses. Consequently, the need for new treatments has arisen. Immunotherapies and targeted therapies have appeared as promising treatments in trials and in clinical settings [1]. Furthermore, combination therapies are starting to be administered, with favorable outcomes in terms of safety and efficacy [1]. 

Immunotherapy is based on a complicated process that includes multiple phases, during which there is a stimulation of the immune system. Consequently, a number of immune cells are transferred to the cancer site with the increase in tumor size and/or growth of new lesions [1].

The staging of melanoma is based on clinical and pathological data described by the staging system of the American Joint Committee on Cancer (AJCC) [1,2]. According to this model, routine imaging is not generally recommended in patients with lower risk (stage I and II) when specific signs or symptoms are absent. However, for clinically node-negative patients, an accurate evaluation of regional lymph nodes should be obtained by employing lymphoscintigraphy (LS) and sentinel lymph node biopsy (SLNB), which remain the gold standards of regional lymph node staging [1,2]. With regard to lymph node assessments with ultrasound (US), this tool shows an overall sensitivity of only 24% for the detection of metastases in SLNs mapped on pre-operative LS [3]. This low rate is due to the inability to detect micrometastases. Several studies have shown that the sensitivity improved with increased cross-sectional area (CSA) of lesion deposits, with a significantly better value when the tumor size exceeded 4.5 mm in diameter [3]. So, pre-surgical US cannot replace SN biopsy in the evaluation of regional lymph nodes [3].

A particular category of lymph nodal metastases is interval or intermediate metastasis, which is characterized by the involvement of non-regional lymphatic stations placed along the pathway of the spread of melanoma, such as the epitrochlear lymph node station, the popliteal fossa, and the retroareolar station [4,5,6,7,8]. The incidence of intermediate metastases in melanoma patients ranges from 3.1% to 7.8%, and, in several patients, these types of lesions could be the only metastatic side [4,7,9,10]. Evidence suggests that the presence of intermediate nodal metastases may represent a negative prognostic feature, since it is associated with an increase in the recurrence and mortality rate [4]. Intermediate metastases should be differentiated from in-transit and satellite metastases, which are both subtypes of superficial metastases. Indeed, metastatic localizations are defined as being “in transit” if they are localized more than 2 cm from the primary melanoma, while they are defined as “satellites” if they are at a distance of less of 2 cm [11]. Our purpose is to report on a comprehensive review of the radiological literature on current radiological data with recent evidence regarding the imaging characteristics and localizations of intermediate metastases.

## 2. Methods

This overview and update is the result of an autonomous study without a protocol or registration number.

### 2.1. Search Criteria 

Several electronic datasets were searched: PubMed (US National Library of Medicine, Bethesda, MD, USA, http://www.ncbi.nlm.nih.gov/pubmed (accessed on 1 May 2022)), Scopus (Elsevier, Alpharetta, GA, USA, http://www.scopus.com/ (accessed on 1 May 2022)), Web of Science (Thomson Reuters, Toronto, ON, Canada, http://apps.webofknowledge.com/ (accessed on 1 May 2022)), and Google Scholar (https://scholar.google.it/ (accessed on 1 May 2022)). The following search criteria were used: “Melanoma”, “intermediate metastases”, “US”, “CT”, “PET-CT”, and “MRI”.

The search covered the years from January 1995 to April 2022. Moreover, the reference lists of the papers found were assessed for papers that were not indexed in the electronic databases. All titles and abstracts were analyzed. The inclusion criteria were clinical studies (e.g., retrospective analyses, case series, prospective cohort studies) evaluating the imaging tools in the assessment of intermediate metastases. Articles published in the English language from January 1995 to April 2022 were included. The exclusion criteria were different topics, unavailability of the full text, insufficient data, or letters to editors.

### 2.2. Results

The search strategy resulted in 6 studies (5 for lymphoscintigraphy and 1 for US and intraoperative lymphoscintigraphy), comprising 18,022 patients, which were further analyzed.

The tumor locations were as follows: epitrochlear lymph node (148), popliteal area (27), peri-umbilical area (10), occipital and postauricular/mastoid areas (12), lateral axillary nodes (3), central axillary nodes (3), triangular inter-muscular space (5), flank (4), peri-areolar area (2), over the deltoid muscle (1), bicipital sulcus (1), cubital nodes (1), subscapular node (1), internal mammary lymph node (2), and aberrant lymph nodes (5). The details of the results are shown in Table 1.

### 2.3. Assessment and Imaging in a Clinical Setting

The localization of intermediate metastases is mainly based on the site of the primary melanoma. In fact, specific drainage patterns have been reported on the basis of the primary lesion [4,12]. For melanomas of the upper extremities, particularly of the ulnar side, the epitrochlear lymph node stations are the typical localizations of intermediate metastases [4,12]. For melanomas of the lower extremities, particularly of the lateral aspect of the heel, the popliteal fossa is a typical site of intermediate metastases [4,12]. For melanomas of the trunk, the triangular intermuscular space and other subcutaneous sites in the back and flank are the most common sites of metastatic localizations, while melanomas on the lateral chest wall are generally drained at the axillary stations [4,12].

Scalp melanomas commonly drain to the occipital or post-auricular/mastoid areas [4,12].

Imaging techniques for the evaluation of patients with melanoma diseases include high-resolution US, computed tomography (CT), magnetic resonance imaging (MRI), and positron emission tomography (PET) [1,10,11,12].

### 2.4. Ultrasound Assessment

Ultrasound is the first choice for the evaluation, staging, and follow-up of patients with melanoma, as it is a non-invasive and inexpensive imaging method, and it shows more sensitivity and specificity than physical examinations [10,13,14,15,16].

In fact, US evaluation allows the detection of metastases that are localized deeper in soft tissues and that are impalpable in clinical examinations [15,16]. Moreover, US can show smaller metastases, which are not well assessed by CT examinations [17].

Sonographic evaluations should be performed with a high-resolution multifrequency linear probe (>7.5 MHz); it would be advisable to have two linear probes with a range of different frequencies available to better evaluate areas of different depths [14,18]. The high frequencies allow the study of superficial areas, while the low frequencies allow the evaluation of deeper lesions (especially in obese patients) and bulky nodal masses [14]. Furthermore, color and power Doppler imaging is essential for the evaluation of intralesional vascular flow; in particular, power Doppler imaging is more sensitive in the evaluation of slower flows [14]. In this regard, the detection of a flow signal when evaluating in-transit nodules is indicative of their solid nature [14,15].

Instead, regarding the use of real-time elastography, some studies have reported the usefulness of this method in the differentiation between reactive and malignant lymph nodes in malignant cutaneous melanoma [19,20]. This examination method involves the exploration of a skin area of at least 10 cm in width around the primary lesion and, subsequently, along the course of the lymph vessels up to the locoregional lymph node station [14].

Sonographic evaluation of the lymph node station should include analysis of the number, the size, the shape, the borders, the internal echo texture, and the hilar structure and distribution [21,22,23].

**Figure 1:**
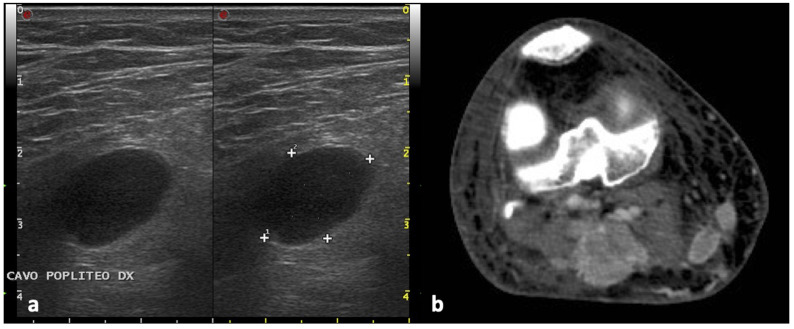
Lymphadenopathy of the right popliteal fossa from cutaneous melanoma of the calf. The B-Mode US scan (**a**) shows an oval, heterogeneous, predominantly hypoechoic lymph node with sharp margins. The contrast-enhanced CT axial-scan image (**b**) demonstrates a partially necrotic lymphadenopathy with peripheral enhancement.

**Figure 2:**
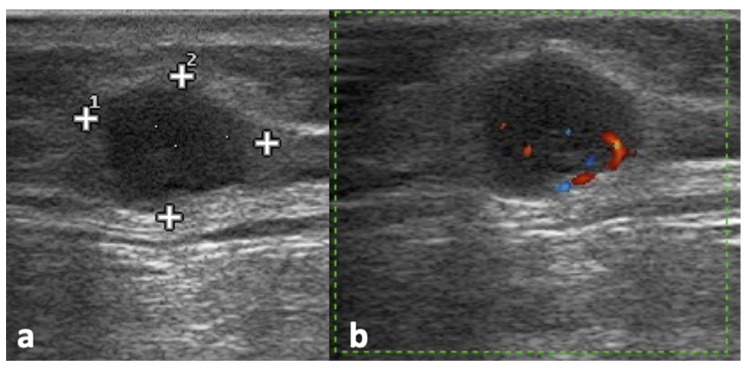
Left subscapular lymphadenopathy representing the recurrence of a cutaneous melanoma on the trunk. The B-Mode US scan (**a**) shows an oval, markedly hypoechoic, inhomogeneous lymph node with irregular borders. The color Doppler scan (**b**) shows prevalent peripheral flow signals.

Regarding the size, lymph nodal measurement should be calculated in two planes to report the maximum diameter and the respective perpendicular diameter (longitudinal and transverse diameters). Regarding the longitudinal diameter, some older studies considered its being greater than 2 or 3 cm as a criterion for suspicion [14,24,25]. Regarding the shape of the lymph node, it should be considered suspicious when it is oval and uniform (Figure 1), and even more suspicious when it is round (Figure 2), while it should not be considered suspicious when it is elongated [14,17,26]. The evaluation of the shape, moreover, should always be integrated with the calculation of the ratio between the longitudinal and transverse diameters (L/T) [14,17,27]. According to some authors, it can be considered suspicious if the L/T ratio is less than 2, while according to other authors, the L/T ratio is suspect when it is less than 1.5 [14,26,27,28]. However, other authors reported that this single criterion is not sufficient for the definition of lymph node malignancy, and it should always be combined with other findings [14].

The borders, instead, may be sharp or irregular [29,30]. Generally, sharp margins are indicative of a reactive lymphadenopathy, while irregular margins are indicative of a metastatic localization; however, this evaluation alone does not allow an adequate differentiation [29,30].

**Figure 3:**
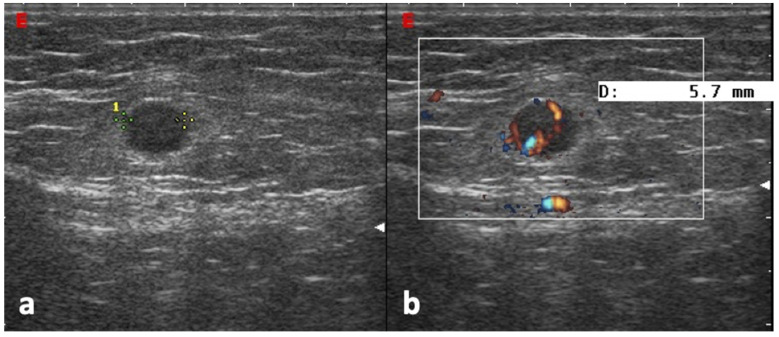
Epitrochlear lymphadenopathy from cutaneous melanoma of the elbow. The B-Mode US scan (**a**) shows a round, hypoechoic, and heterogeneous lymph node with irregular borders. The color Doppler scan (**b**) shows an intense and anarchic vascularity.

Regarding the evaluation of the internal echostructure, a typical finding is that a metastatic lymph node has a markedly and diffusely hypoechoic echo texture (Figure 1, Figure 2 and Figure 3) [14]. However, some authors considered the importance of the examination of cortical morphological abnormalities, as they may indicate an early stage of lymph node metastasis, possibly preceding the other typical features [14]. In this regard, it is well known that cancer cells transmitted by lymphatic vessels reach and grow first within the cortex and then in the rest of the lymph node [31]. Consequently, cortical thickening, which can be circumferential (symmetric), unilateral (asymmetric), or focal (possibly nodular), may representant an early finding [31]. Unilateral cortical thickening should be considered much more suspicious than circumferential thickening, while nodular thickening should be clearly interpreted as metastatic [31]. Nodular thickening, also called a “nodule within the node”, can be isoechoic or hypoechoic to the rest of the cortex; in both cases, it indicates a metastatic localization [14,31]. Moreover, another suspicious cortical abnormality may be a focal bulging or protuberance [31].

Regarding color and power Doppler sonography evaluation, in metastatic lymph nodes, the hilar region, which is normally centrally localized, may be dislocated or small in size, and may even disappear (Figure 2) [32].

Other vascular signal features to be evaluated include intensity, origin, and distribution [21].

The intensity can be increased in metastatic lymph nodes; however, this finding is not very specific, as it can also be present in benign lymph nodes [21].

The origin is typically hilar in benign lymph nodes, with monopolar vascularity; in malignant lymph nodes, multiple vessels can penetrate the lymph node through the capsule [32,33,34].

The distribution is generally homogeneous in benign lymph nodes, while it is inhomogeneous or anarchic in malignant lymph nodes [14,33,35].

Therefore, the combination of US findings, such as round or oval morphology, markedly hypoechoic structure, focal or nodular and asymmetric cortical thickening, and hilum loss or dislocation with anarchic distribution of the vascular signal, regardless of size, is virtually diagnostic of metastasis [14,28,34,35,36,37,38,39,40,41].

### 2.5. Assessment with Other Imaging Techniques (CT, MRI, PET)

Regarding the use of different imaging techniques for intermediate melanoma metastases, each method has advantages and disadvantages [42,43,44].

CT is currently the most widely used imaging technique for melanoma staging and follow-up [13,45,46,47,48,49,50,51]. PET allows a functional study, as well as a whole-body evaluation, in a single scan [13,45,46].

In CT evaluation, there are no features that reliably indicate metastatic lymph node involvement. The only reliable morphological feature is the size; in fact, an increase in nodal size compared to the previous examination is generally indicative of malignancy [13,45,46].

MRI shows superior accuracy, sensitivity, and specificity to those of CT when detecting lymph nodes ranging in size from 1 to 5 mm, without using ionizing radiation [52,53,54,55,56,57,58].

A metanalysis assessed the roles of four imaging methods (US, TC, PET, and PET–CT) in the staging and follow-up of melanoma patients, and it showed that ultrasonography had the highest sensitivity (60%, 95% CrI = 33% to 83%), specificity (97%, 95% CrI = 88% to 99%), and diagnostic odds ratio (42, 95% CrI = 8.08 to 249.8). For staging of distant metastases, PET–CT had the highest sensitivity (80%, 95% CrI = 53% to 93%), specificity (87%, 95% CrI = 54% to 97%), and diagnostic odds ratio (25, 95% CrI = 3.58 to 198.7). Similar trends were observed for the surveillance of involvement of lymph nodes in melanoma, with ultrasonography having the highest sensitivity (96%, 95% CrI = 85% to 99%), specificity (99%, 95% CrI = 95% to 100%), and diagnostic odds ratio (1675, 95% CrI = 226.6 to 15,920). For distant metastases, PET–CT had the highest sensitivity (86%, 95% CrI = 76% to 93%), specificity (91%, 95% CrI = 79% to 97%), and diagnostic odds ratio (67, 95% CrI = 20.42 to 229.7). Positive predictive values were likewise the highest for ultrasonography in lymph node staging and for PET–CT in detecting distant metastases [59].

## 3. Discussion and Conclusions

The evaluation of lymph node metastases represents a fundamental point in the staging and follow-up of melanoma. In fact, detection of interval metastases has a crucial role in the management of patients with melanoma, as it has a negative prognostic role associated with an increase in the recurrence and mortality rates [4]. In the case of a negative lymph node biopsy, the patient is staged at level I or II [13]. Instead, a positive lymph node biopsy is indicative of clinical stage III disease and requires baseline imaging to detect the possibility of clinically occult stage IV disease [13].

The usefulness of imaging studies in patients with melanoma generally depends on the stage of the disease. In patients with early-stage disease, surgery is often curative, and, generally, the most commonly used preoperative imaging methods for the evaluation of regional nodal drainage, as well as potential alternative or unpredictable nodal drainage basins, are ultrasound and/or lymphoscintigraphy [13].

In patients with stage III and IV disease, the imaging techniques performed are a contrast-enhanced whole-body CT scan or PET–CT [13]. However, superficial lymph node stations, i.e., the intermediate and in-transit stations, are difficult to detect with CT and MRI, which is mainly due to their small size, while they are more easily detectable in clinical examinations and with US [13].

Therefore, a thorough knowledge of the imaging methods available and the interactions between the clinician and the radiologist are essential for making the correct choices for individual patients, for better management, and to improve treatment and survival [60,61,62,63].

Although US is non-invasive, it involves costs and sophisticated machines. In addition, a high expertise of the physician is mandatory to recognize the normal structures of the lymph nodes draining the lesion. The first sign of metastasis in an SN is habitually identified in the sub-capsular sinus at the point of entry of the afferent lymphatic that drains the primary melanoma. An early metastasis in the sub-capsular sinus is an elongated tumor cell aggregate. Several lesions with a low CSA are not detectable when utilizing the existing technologies. However, in these situations, it is possible to evaluate indirect signs of metastasis, such as an increase in the vascular signal. This feature can be detected by using color Doppler sonography, although other pathological conditions could cause an increase in blood flow in this site. However, US is more sensitive and specific than physical evaluation, and, with respect to other diagnostic tools, such as CT or PET–CT, it is superior for detecting lymph node metastases during surveillance.

Regarding differential diagnoses, many conditions can be associated with the presence of a nodular image within soft tissues, including normal or abnormal vessels, dense scars, and nodal and extra-nodal diseases [34,64,65,66]. Nodal causes include acute lymphadenitis (e.g., cat scratch disease), tubercular lymphadenitis, sarcoidosis-related lymphadenitis, lymphomas, and metastatic lymphadenopathies (especially from melanomas, but also from other cutaneous and non-cutaneous cancers) [34,64,65,67]. Extra-nodal causes include cysts, fluid collections (seromas, hematomas, and lymphoceles), abscesses, tumors (nerve tumors, fibromas, hemangiomas, lipomas, and Merkel cell tumors), cutaneous and subcutaneous hematogenous metastases, and Kimura’s disease [22,32,34,64,65,67,68,69,70,71,72,73].

However, the combination of the patient’s history information with features of B-mode and color/power Doppler US usually allows an adequate differential diagnosis [14,22,34].

In conclusion, a thorough knowledge of the main localizations, findings, and imaging methods for intermediate metastases is necessary for better management and to improve the treatment and survival of patients with melanoma.

## Figures and Tables

**Table 1:** Assessed studies: number of patients; tumor locations; SNL locations.

	Uren et al. [12]	Hunt et al. [5]	Uren et al. [7]	Roozendaal et al. [9]	Ishihara et al. [6]	Mcmasters et al. [4]
**Year**	1995	1998	2000	2001	2003	2020
**Number of Patients**	450	13,139	2045	379	9	2000
**Tumor location**						
***head and neck***			304	35		219
***trunk***			905	133		901
***lower extremities***			451			457
leg or foot				153		
***upper extremities***			385			423
arm				58		
upper arm					2	
forearm/elbow		700			1	
wrist						
hand		102			1	
fingers					5	
**SLN location**			148			
epitrochlear lymph node		10		2		15
popliteal area				3		8
peri-umbilical area	10					
occipital and postauricular/mastoid areas						12
lateral axillary nodes					3	
central axillary nodes					3	
triangular inter-muscular space				5		
flank				4		
peri-areolar area				2		
over the deltoid muscle				1		
bicipital sulcus				1		
cubital nodes					1	
subscapular node					1	
internal mammary lymph node	2					
aberrant lymph nodes				4	1	

## Data Availability

Data are available at https://zenodo.org/record/6693317#.YrS913ZBy3A (accessed on 1 May 2022).
